# Degradation Mechanism of a Sauce-Glazed Ware of the Song Dynasty Salvaged out of the Water at Dalian Island Wharf: Part I—The Effect of the Surface-Attached Composite Coagula

**DOI:** 10.3390/ma16031176

**Published:** 2023-01-30

**Authors:** Rao Ding, Weidong Li, Zelin Yang, Changsong Xu, Xiaoke Lu

**Affiliations:** 1 Shanghai Institute of Ceramics, Chinese Academy of Sciences, Shanghai 201899, China; 2 Key Scientific Research Base of Ancient Ceramics, State Administration for Cultural Heritage, Shanghai 201899, China; 3 Key Laboratory of the Comprehensive Analysis Technology for Ancient Ceramics and Its Applications, Ministry of Culture and Tourism, Shanghai 201899, China; 4 University of Chinese Academy of Sciences, Beijing 100049, China; 5 Institute of Cultural Relics and Archeology, Fujian Museum, Fuzhou 350025, China

**Keywords:** sauce-glazed ware, degradation mechanism, microstructure, cellular coagula, coralline algae, inorganic pollutants

## Abstract

Dalian Island is located in the sea area near Pingtan County, Fujian, Southeast China. The sea area used to be the junction of the eastern and western ship routes on the Maritime Silk Road, and is also an important region for underwater archaeology in China. This study focused on a sauce-glazed ware of the Song Dynasty, with serious degradation, which was salvaged out of the water at the Dalian Island Wharf. Optical microscopy, scanning electron microscopy, X-ray diffraction analysis, and micro-Raman spectroscopy were used to comprehensively analyze the composition, phase attributes and microstructure of the ware and the surface-attached coagula. The findings revealed that the sea wave-borne debris scoured the surface of the ware, causing mechanical damage to varying degrees and a significant decrease in its degradation resistance. This was the primary factor accounting for the poor preservation state of the salvaged ceramic ware, and the precondition for the subsequent attachment of marine organisms and the deposition of inorganic pollutants. The calcareous skeletons formed on the surface induced by the bio-mineralization of coralline algae (a type of marine plant) could resist the mechanical action caused by the motion of sea waves, thereby slowing down the ware’s degradation process. In other words, the calcareous skeletons played a ‘bio-protective’ role to a certain degree. In addition, inorganic pollutants represented by iron rusts also participated in the corrosion of the glaze. Some pollutants were directly deposited on the pits and cracks on the surface of the ware, which brought stress to the glaze and glaze/body interface, causing the glaze to further crack and spall. Moreover, iron rusts reacted with the glaze, leading to chemical alteration, accompanied by the formation of iron silicate as the alteration product. Anorthite crystals in the interlayer did not participate in the reaction but remained at the original position. The alteration product gradually replaced the original glass phase of the glaze and entered into the body via pores and cracks. In conclusion, the complex degradation morphology of the salvaged sauce-glazed ware could be attributed to the combined action of mechanical damage, marine bio-fouling, and chemical alteration.

## 1. Introduction

The rapid development of underwater archaeology in China has facilitated the discovery of a large number of trade shipwrecks on the Maritime Silk Road. Ceramic wares, as important goods in ancient China, play a dominant role in the relics salvaged out of water [1]. Salvaged ceramic wares, characterized by wide varieties and abundant kiln sites, not only reflect the achievements of ancient Chinese exported ceramics in manufacturing technology and the style of the times, but also serve as the exchange carrier of culture and technology. The preservation conditions of the salvaged ceramic wares are closely associated with the marine environment. Ceramic wares are brittle and hence suffer from breakage under mechanical force, which is the most common irreversible damage. Seawater is a kind of complex chemical system containing multiple substances such as water, inorganic salt, and soluble gas. When buried in seawater (a natural strong electrolyte solution), the liquid-solid interfacial chemical reaction can occur on the surface of ceramic wares, leading to the corrosion of glaze and discoloration of over-glaze patterns [2,3]. After being filled with soluble salt, the open holes of the body may undergo the solution–crystallization–resolution–recrystallization process and volume change, which can cause considerable seepage stress [4]. The desalination methods for salvaged ceramic wares include soaking in deionized water, ultrasonic cleaning, and high-temperature cleaning [1,5,6].

Wrapping and cementation by coagula are common phenomena of salvaged ceramic wares. Hard and firm coagula are problems in the further preservation of cultural relics. Researchers investigated the chemical compositions of the coagula on the surface of salvaged ceramic wares and found that the skeletal remains of marine organisms constituted the main calcium compositions in the coagula [3,7]. Moreover, ferrous coagula were primarily composed of hematite, magnetite, goethite, and pyrite, and mainly sourced from iron corrosion products in seawater [8,9,10]. Additionally, siliceous coagula were mainly sourced from seabed clay minerals and sea sands [7]. For different types of coagulum, the corresponding removal method has been adopted [3,10,11,12,13]. Researchers have also proposed several methods for evaluating the possible damage from conservation measures for salvaged ceramic wares [14,15,16].

The heterogeneity of glaze is closely related to the chemical alterations under a marine or soil environment. For example, the preferential corrosion of anorthite in the crystallization-phase separation glazes is the main important reason for serious corrosion [17,18,19].

At present, the research on the formation mechanism of coagula is insufficient. In particular, the effect of marine organism attachment on the degradation of ancient ceramic wares has not been reported. In this study, a salvaged sauce-glazed ware of the Song Dynasty was comprehensively analyzed using multiple methods. On that basis, the degradation mechanism of the ware in the marine environment was concluded. This paper reports Part I of the degradation mechanism of the salvaged sauce-glazed ware. In Part I, the complex coagula composed of the attached coralline algae, iron rusts, and sea mud were investigated. Part II of the study will be reported in a follow-up paper, which focuses on the surface-attached marine animal remains.

## 2. Sample

**Figure 1:**
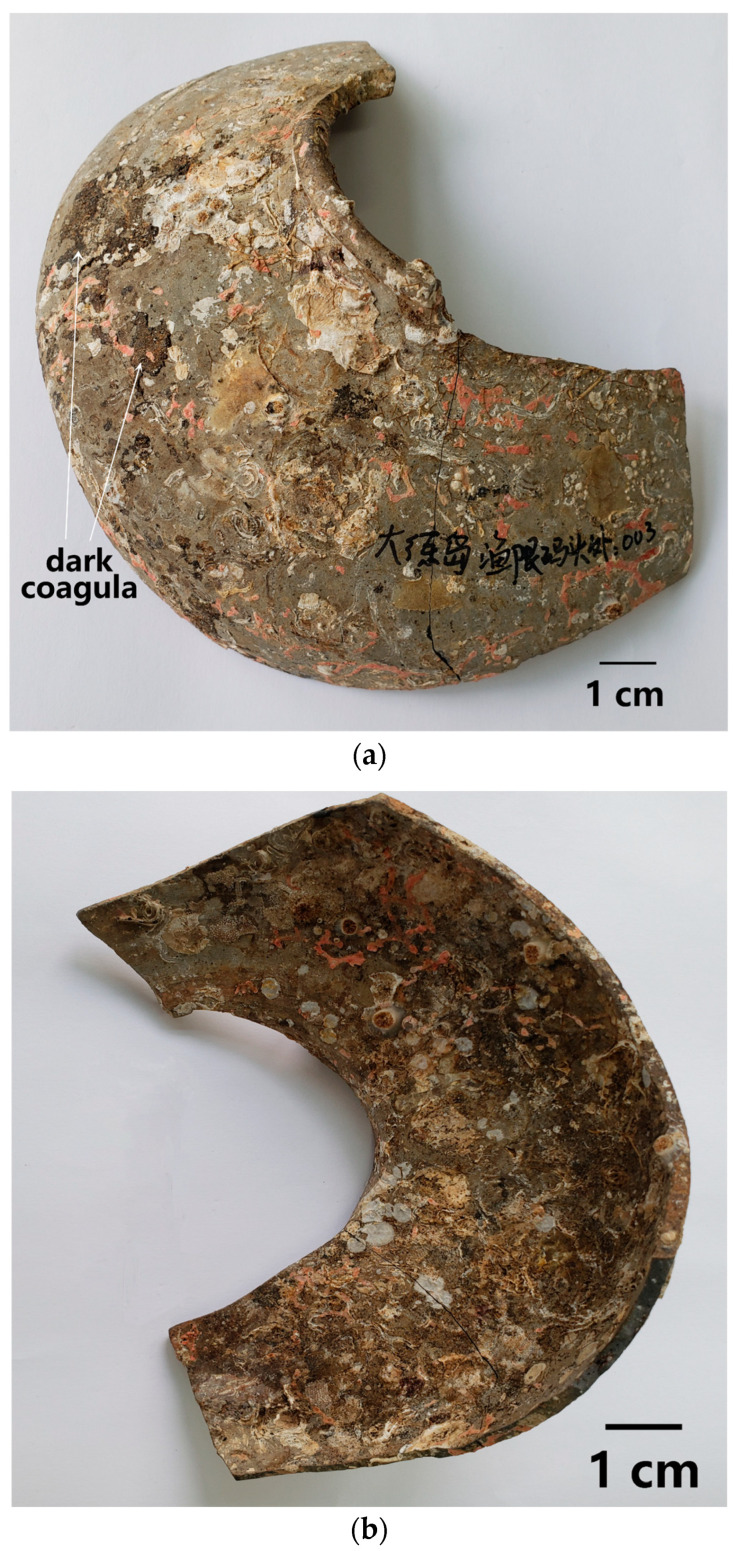
Appearance of the sauce-glazed shard DL of the Song Dynasty salvaged out of water at the Dalian Island Wharf: (**a**) outer surface, showing serious glaze spalling, dark coagula, and various marine animal remains; (**b**) inner surface, showing contamination and various marine animal remains. Chinese in (**a**): out of the Dalian Island Yuxian Wharf: 003.

In this study, a sauce-glazed ware, numbered DL, was salvaged from the Dalian Island Wharf, Pingtan County, Fujian, China. The visual inspection revealed that it was the relic of a sauce-glazed pot produced in the Song Dynasty. The glaze on the outer surface of the ware was seriously spalled (Figure 1a), with large-area exposure of the body. The inner surface was not glazed (Figure 1b). Complex and diverse coagula were distributed on both the outer and inner surfaces. The shard DL was cut at a position with dark coagula attached to the outer surface to obtain a small sample DL-1, and was cut at two positions with serious spalling of glaze to obtain samples DL-2 and DL-3. After coarse and fine grinding, the cross-sections of the samples DL-1~3 were polished by 1500 mesh boron carbide micro-powder. After this, the samples were cleaned with deionized water twice and absolute ethyl alcohol once, with a duration of 15 min each time. Finally, the samples were placed in the blast air oven and dried at 110 °C for 3 h.

## 3. Research Method

The morphology of the natural surface and polished cross-sections of the samples were observed by an optical microscope (OM, Keyence VHX-2000, Osaka, Japan), using halogen lamp (12 V/100 W) as illumination source, with scanning frequency of horizontal (H): 75 kHz, vertical (V): 60 Hz, CCD image sensor with total pixels of 1688 (H) × 1248 (V), effective pixels of 1600 (H) × 1200 (V). The microstructure and semi-quantitative micro-area chemical compositions of the natural surfaces and polished cross-sections of the samples were analyzed by a field-emission scanning electron microscope equipped with energy dispersive spectrometer (SEM-EDS, FEI Magellan 400, Waltham, MA, USA), using extra high tension of 15–20 kV, probe currents of 0.1–6.4 nA, resolution < 0.8 nm at 15 kV. Before the SEM-EDS analysis, natural surfaces and polished cross-sections of the samples were coated with carbon. The number of acquisitions for EDS analysis was once.

The chemical compositions of the samples were analyzed using an energy-dispersive X-ray fluorescence spectrometer (EDXRF, Bruker M4 TORNADO, Germany). During the observation, the power of the X-ray light tube was 30 W (with a voltage of 50 V and a current of 600 μA), and the diameter of the X-ray focus was 25 μm.

The coagula were analyzed using a microscopic Raman spectrometer (µ-Raman, Horiba XploRA ONE, Kyoto, Japan), with the Raman spectral signal ranging from 100 cm^−1^ to 3500 cm^−1^ and an excitation wavelength of 532 nm. The confocal microscope (Olympus BX41) was employed to focus the laser beam on the test areas with ×10 and ×50 objectives.

The phase attributes of the coagula were identified by an X-ray diffractometer (XRD, Bruker D8 Discover, Germany) using Cu Kα radiation (40 kV, 40 mA). The step size was selected to be 5°/min with 2θ values ranging from 10° to 80°.

Sample DL-1 was analyzed by μ-Raman, XRD and EDXRF. Samples DL-1~3 were analyzed by OM and SEM-EDS. All the analyses were carried out on natural surfaces or polished cross-sections.

## 4. Experimental Results

### 4.1. Chemical Composition of the Body and Glaze

**Figure 2:**
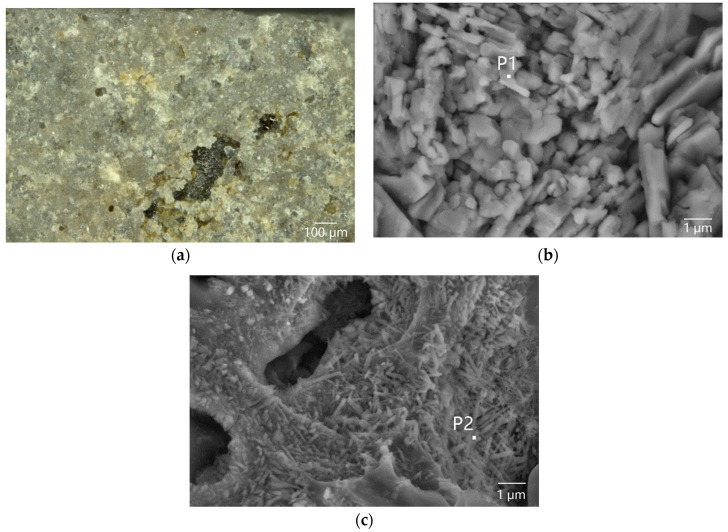
Morphology of the outer surface of sample DL-1: (**a**) OM image, showing the naked body at the local spalling position of the glaze; (**b**) SEM image, showing the rod-like anorthite crystals in the body/glaze interlayer exposed from the breakage of the glaze; and (**c**) SEM image, showing the sill-like crystal clusters composed of mullite crystals.

The residual glaze on the outer surface and the fresh cross-section of the body were analyzed by using EDXRF, as listed in Table 1. In the body, the contents of Si and Al were 64.59 wt% and 17.47 wt%, respectively. Accordingly, the body could be classified as a high-silicon low-aluminum type of south China. The glaze showed extremely unreasonable chemical composition data. To be specific, the contents of Mg and Fe were up to 7.07 wt% and 38.44 wt%, respectively. The data were closely correlated with the pollution and alteration of the glaze. Foreign pollutants carried by seawater were gradually deposited on the glaze and simultaneously invaded into the glaze via cracks and porous channels (Figure 2). 

### 4.2. Morphology and Microstructure of the Samples

The OM observation (Figure 2a) showed that the glaze on the outer surface of sample DL-1 was rough and contained a lot of microcracks. The SEM observation and micro-area EDS analysis of sample DL-1’s surface (Table 2) confirmed that the body/glaze interlayer exposed at the breakage position of the glaze was composed of rod-like anorthite crystals (Figure 2b and P1 in Table 2). The sill-like crystal clusters composed of needle-like mullite crystals were found in the naked body, which were transformed from the clay minerals in the body at approximately 1000 °C (Figure 2c and P2 in Table 2) [20].

**Figure 3:**
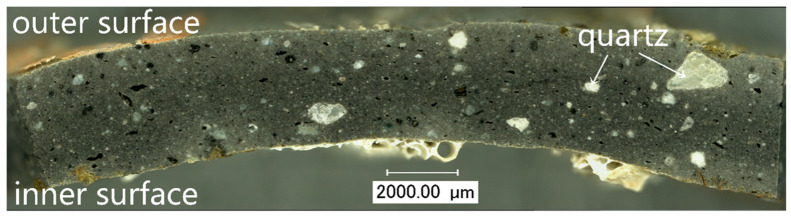
OM image of the cross-section of sample DL-2, showing a lot of quartz particles in the body.

The OM image of the cross-section of sample DL-2 showed that the body was featured by a lot of pores. A large number of quartz particles of different sizes, with a maximum diameter of up to approximately 0.2 mm, were found in the body, as shown in Figure 3. It could be inferred that the quartz particles were intentionally added for enhancing the resistance to thermal distortion [20]. During the mixing of raw materials, the quartz particles of different sizes were wrapped with iron-bearing clay. In the following firing and cooling process, the quartz underwent an α–β phase change at 573 °C twice [4]. The cracks then appeared because of repeated volume expansion and shrinkage.

**Figure 4:**
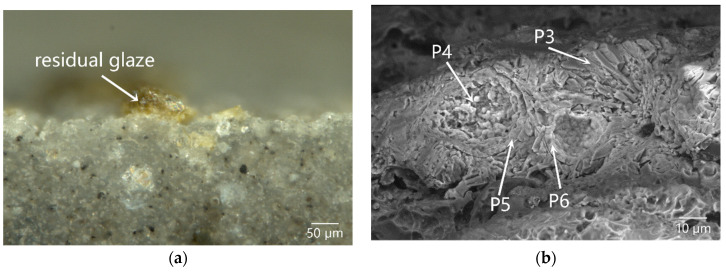
Cross-sectional morphology of the residual glaze on the outer surface of sample DL-2: (**a**) OM image and (**b**) SEM image, showing foreign pollutants deposited in the pores in the residual body/glaze interlayer.

Figure 4a showed a magnified OM image of a cross-section of DL-2 at a position with residual yellow-green glaze on the outer surface. Based on the SEM image, most of the glaze was spalled, and only part of the anorthite interlayer remained, as shown in Figure 4b. The EDS data indicated that the content of Mg of the residual interlayer was as high as 13.54 wt% (P3 of Table 2), which could be attributed to the deposition of Mg^2+^ from the seawater at the aforementioned position. As shown in Figure 4b, two circular pores, with a diameter of approximately 30 µm and 20 µm, respectively, could be observed in the residual interlayer. The holes were surrounded by anorthite crystals. A layer of foreign pollutants mainly composed of Fe, Si, Mg, and Al was deposited on the inner surface of the pores, as listed in P4, P5, and P6 of Table 2, in which the content of Fe reached up to 37–65 wt%. The two pores were originally two big bubbles discharged from the glass phase in the interlayer. When the glaze was destroyed by the external mechanical force, the pores changed from closed-type to open-type in the glass phase, and were finally directly exposed to the marine environment, leading to the invasion by foreign pollutants. The foreign pollutants might be a mixture of iron rusts, sea sands, and sea mud.

**Figure 5:**
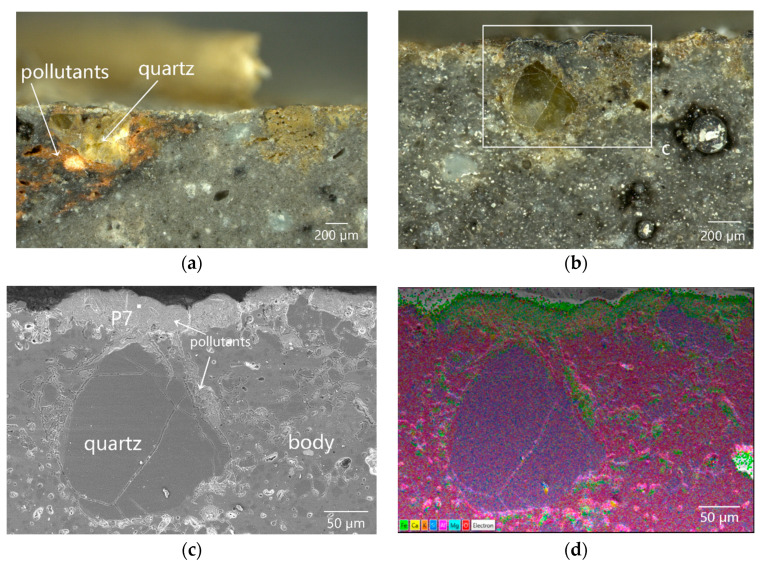
Cross-sectional morphology of sample DL-3: (**a**,**b**) OM images of quartz particles exposed to the surface of the inner surface of the body, (**c**) SEM image of the enlarged image of the region in the box of (**b**), and (**d**) element mapping image of (**c**).

The quartz partly exposed to the surface of the inner surface suffered from foreign pollution. The OM observation showed that the cracks around the quartz provided the channels for the invasion of pollutants into the body. The brown pollutants entered into the interconnected pores and cracks of the body along the surrounding cracks of the quartz and extended as far as a depth of approximately 1 mm from the surface of the body (Figure 5a).

The SEM and EDS element mapping of the polluted quartz section (Figure 5b) showed that the foreign pollutants with different contrast degrees were filled into the microcracks between the quartz and the body, and gradually deposited on the surface of the inner surface (Figure 5c). The foreign pollutants, mainly composed of Fe, Si, Mg, and Al (Figure 5d and P7 in Table 2), were identical to the foreign pollutants shown in Figure 3 in terms of sources, which were the mixture of iron rust, sea sands, and sea mud.

**Figure 6:**
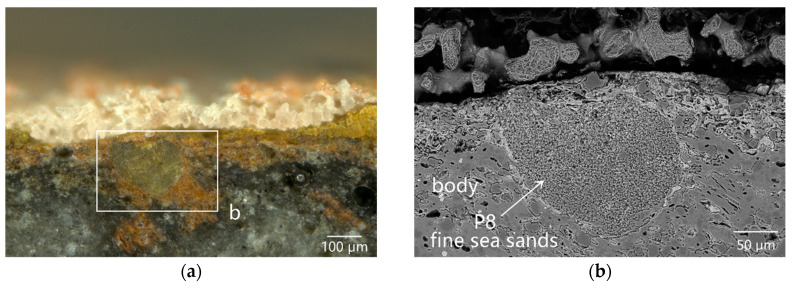

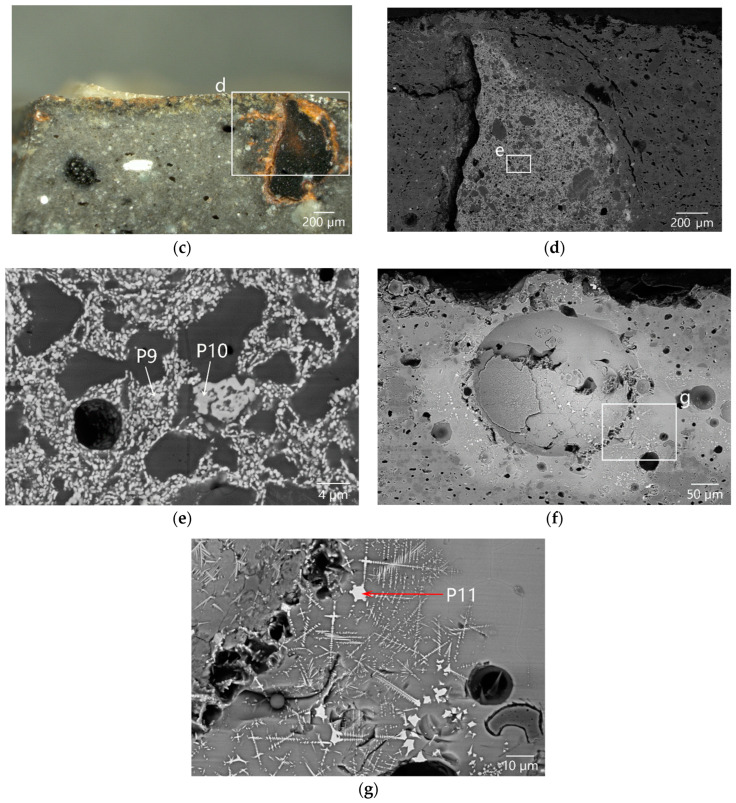
Cross-sectional morphology of sample DL-3: (**a**) OM image showing that an open pore on the surface of the inner surface was polluted by iron rusts, then filled with fine sea sands, and finally covered by red coral skeletons; (**b**) SEM image of the enlarged image of the box in (**a**); (**c**) OM image of the black blocks around the inner surface of the body; (**d**) SEM images of the enlarged image of the box in (**c**); (**e**) SEM image of the enlarged image of the box in (**d**) showing a large number of quartz particles in the black blocks wrapped with iron oxides and rutile microcrystals; (**f**) SEM image of the large bubble near the body/glaze interface; and (**g**) SEM image of the enlarged image of the box in (**f**), showing that a lot of iron oxide dendrites were precipitated from the iron-rich glass phase around the large bubble.

The exposed open-type pores or crevasses on the inner surface of the body, formed due to mechanical damage, could provide sites for the deposition of fine sea sands. As shown in Figure 6a, an open-type pore on the surface of the inner surface of sample DL-3 was polluted by iron rusts. Obviously, the iron rusts with shallow contrast degrees were observed in the extended cracks. The rusts penetrated into the deep via the cracks, and simultaneously, the pores were filled with fine sea sands (as illustrated by EDS data in P8 of Table 2) and then covered by red coral skeletons (Figure 6a).

Large brown-black blocks could also be observed on the surface of the inner surface. The surrounding cracks, with a diameter of 50–80 µm, were slightly filled with yellow pollutants (Figure 6c). The SEM image in Figure 6d showed a lot of small quartz particles with microcracks and densely distributed high-contrast microcrystals. The quartz particles were mostly less than 40 µm in size. The crystals were hexagonal iron oxide crystals with a size of less than 1 µm and short-column rutile crystals with a length of less than 5 µm (Figure 6e and EDS results in P9 and P10 in Table 2).

A bubble with a diameter of 300 μm was found near the body/glaze interface, and a lot of dendritic crystals precipitated from the surrounding glass phase (Figure 6f). The formation of the bubble could be attributed to the fact that the gas generated from the hydrolysis of iron oxide in the raw material could not be discharged to the outside in time. As the bubble rose, more iron was brought to near the body/glaze interface. During the cooling process, a lot of iron oxide dendrites were precipitated from the iron-rich glass phase around the bubble (Figure 6g; EDS data in P11 of Table 2). The EDS data in P8–P10 of Table 2 were significantly affected by a large beam spot and X-ray penetration.

### 4.3. Analysis Results of the Cellular Coagula Attached to the Outer Surface of the Samples

#### 4.3.1. Morphology of the Cellular Coagula

**Figure 7:**
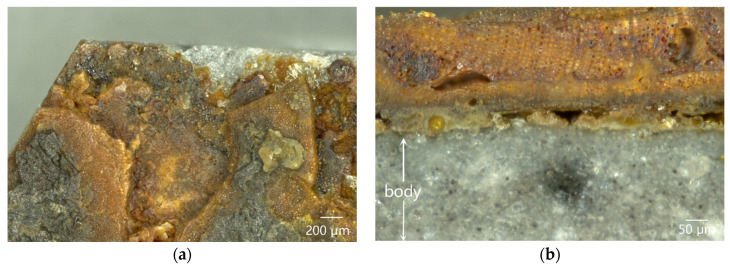
OM images of cellular-structure coagula attached to the outer surface of sample DL-1: (**a**) surface and (**b**) cross-section.

Both surface and cross-section of the dark coagula attached to the outer surface of sample DL-1 were observed under the OM, as shown in Figure 7. In Figure 7a, the coagula exhibited quite nonuniform distributions in both color and quality, with obvious heterogeneity. Figure 7b showed the structure of the dark coagula observed on the polished cross-section. Cellular structures with regular arrangement could be seen in the coagula. Moreover, residual glaze was observed between the coagula and the body, with a maximum thickness of only approximately 50 µm. The damage and thinning of the glaze suggested that the ware was impacted and abraded by sea wave-borne debris before the attachment of coagula. The thickness of the cellular-structure coagula was more than 200 µm. The existence of coagula prevented the ware from being further abraded, so that the residual glaze could have been retained.

#### 4.3.2. Microstructure and Microarea Composition of the Coagula

**Figure 8:**
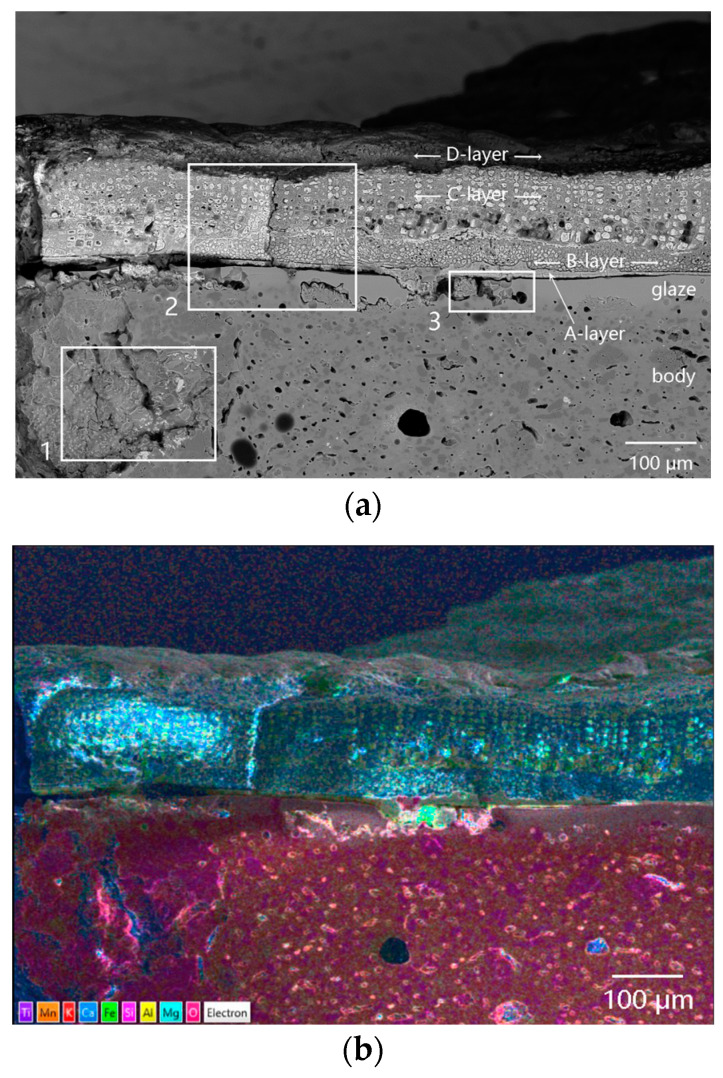
Cross-sectional morphology of the cellular-structure coagula attached to sample DL-1: (**a**) SEM image and (**b**) element mapping images.

**Figure 9:**
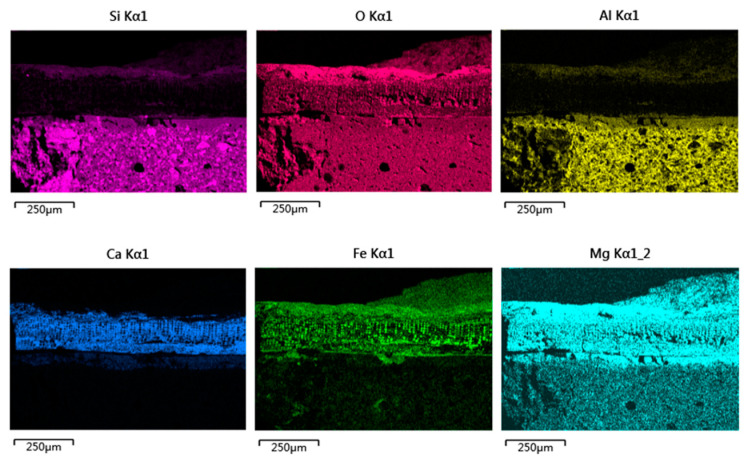
Element mapping images of sample DL-1 with cellular coagula attached.

**Figure 10:**
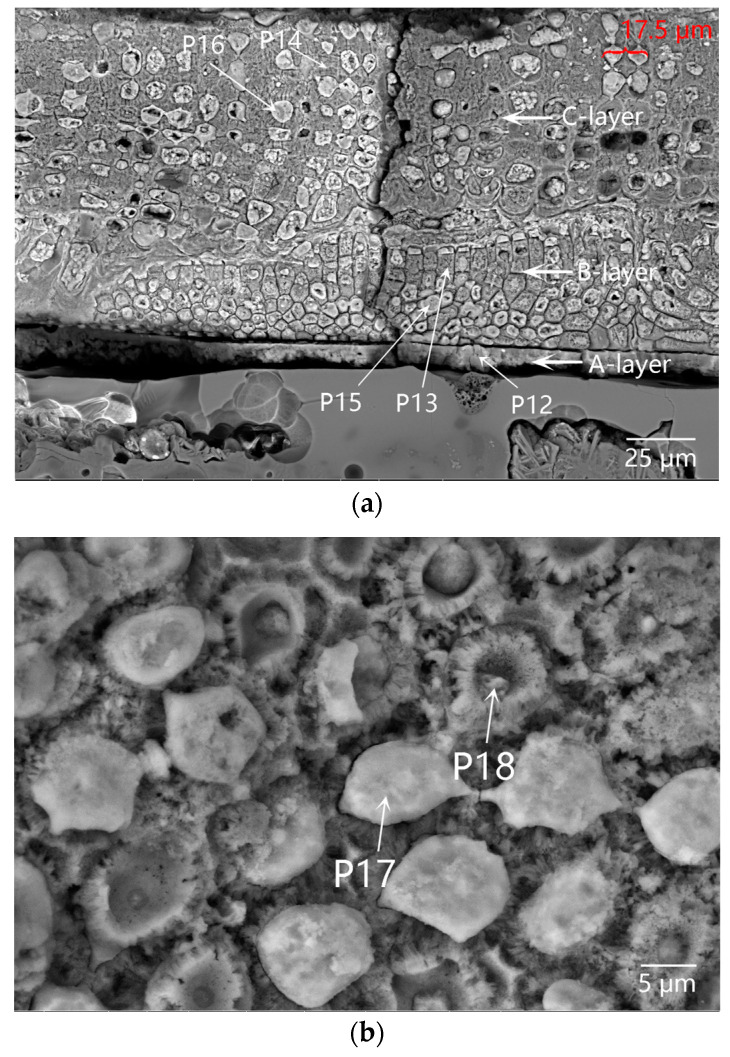
SEM images of the cellular-structure coagula attached to the outer surface of sample DL-1: (**a**) coagula (A-layer), coralline algae skeleton and filler (B-layer and C-layer) in the No. 2 box of Figure 8a, observed upon the cross-section; and (**b**) coralline algae skeleton and filler observed upon the surface.

Figure 8 showed the SEM images and element mapping images of the cellular-structure coagula. A lot of quartz and a few iron-bearing mineral particles were found in the body. As shown in the No. 1 box of Figure 8, the body was locally broken under mechanical force. Figure 8a showed that residual glaze/body interlayer existed, and the cellular-structure coagula showed clear stratified structures. The A-layer coagula, located between the cellular coagula and the glaze (at the center of No. 2 box in Figure 8a). The combined results of the element mapping (Figure 9) and micro-area EDS data(P12 in Table 2) revealed that A-layer were mainly composed of Fe and Si (Figure 10a). In particular, the content of Fe was as high as 62.44 wt%. The No. 2 box showed that the cracks throughout the cellular coagula were exactly located above the A-layer coagula, which suggested that some pollutants, including iron rusts and sea sands, were driven by seawater and carried to the glaze surface via the cracks.

The B-layer and C-layer coagula showed similar morphology, which were overall composed of a cellular skeleton and the foreign substances filled in the porous channels (on the top of No. 2 box in Figure 8a and local map in Figure 10a). The porous channels were less than 10 µm in diameter. Part of the fillers fell off from the channels during the sample cleaning process. The B-layer coagula were relatively thin, with a thickness of 20–40 µm. By contrast, the thickness of the C-layer coagula was up to 80 µm. The diameter of pore channels in the B-layer was smaller than that in the C-layer. The combined results of the element mapping (Figure 9) and micro-area EDS data revealed that the cellular skeleton differed significantly from the filler in terms of chemical composition. The cellular skeleton was rich in Ca and Mg (P13 and P14 of Table 2), and the filler was rich in Fe and Si (P15 and P16 of Table 2). The filler was highly similar to the A-layer coagula (P12 in Table 2) in chemical composition. Contrary to the B-layer skeleton, the C-layer skeleton showed higher content of Ca but lower content of Mg.

Referring to the published SEM images illustrated in Ref. [21], the cellular skeleton attached to the outer surface of the sample was confirmed as the remains of coralline algae (a kind of marine benthos). The diameter of a single cell of coralline algae was approximately 10 µm, as shown in Figure 10a. During bio-mineralization, coralline algae secreted calcium carbonate on the cell walls in the form of calcites, and dissolved different amounts of magnesium in the crystal lattice [21]. The pile-up of solid calcareous cells formed the B-layer and C-layer cellular matrices, which were then referred to as the medulla and cortex of coralline algae in biology. The B-layer and C-layer matrices were also called hypothallium and perithallium, respectively, with different cell arrangement patterns. According to highly similar chemical compositions between the filler and A-layer coagula, it was inferred that the filler was deposited by foreign inorganic pollutants in the porous channels left by the cytoplasmic degradation of the coralline algae.

The outermost layer, that is, the D-layer coagula, with a thickness of 20–50 µm, was mainly composed of Si, Al, Fe and Mg, without Ca (the single-element mapping in Figure 9). This was attributed to the deposition of some foreign pollutants such as iron rusts, sea sands, and sea mud.

**Figure 11:**
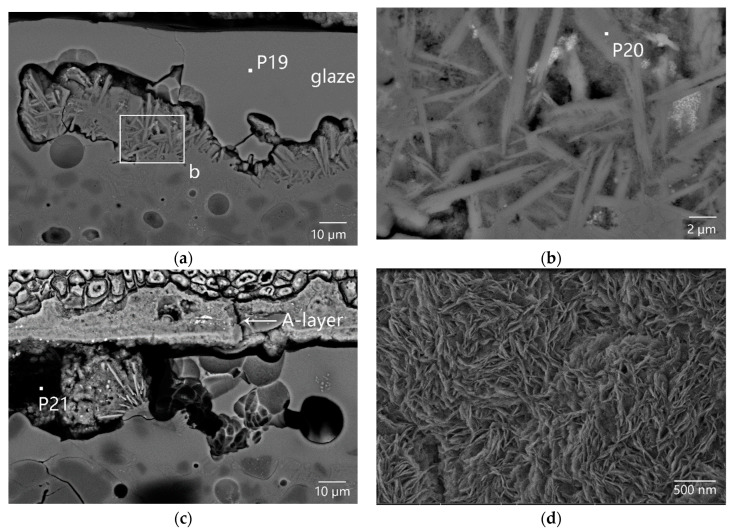
Cross-sectional SEM image of the coagula/glaze interface in sample DL-1: (**a**) at the bottom of No. 2 box in Figure 8a, in which the residual glaze and the body/glaze interlayer were observed, (**b**) rod-like anorthite crystals in the body/glaze interlayer, (**c**) No. 3 box of Figure 8a, in which the broken glaze between the cellular coagula and the body were observed, and (**d**) morphology of the foreign pollutants deposited in the pore at P21.

Crustose coralline algae attached to the outer surface of sample DL-1 grew in a covering pattern. Microcracks gradually formed on calcareous algae skeletons under external mechanical force, as shown in Figure 10a. Both microcracks on the coralline algae skeletons and porous channels formed after the degradation of cytoplasm provided foreign pollutants with invasion paths. Figure 10b showed the SEM image of the cross-section of cellular coagula from the top view. The porous channels after the degradation of coralline algae cytoplasm at P17 were completely filled by the filler. At P18, the pore was partially filled. According to the EDS data, the filler at P17 in Table 2 was almost identical to the filler at P15 and P16 in Table 2 in terms of chemical composition. The filler was mainly composed of Fe and Si and sourced from foreign pollutants, including iron rusts, sea sands, and sea mud. The filler at P18 was mainly composed of Fe and Ca and sourced from the filler and calcareous algae skeleton. The residual glaze below the calcareous algae remains (the bottom of No. 2 box in Figure 8a and Figure 11a), with a thickness of 50–60 µm, could be regarded as a sauce glaze according to EDS data (P19 in Table 2). The data were more reasonable than the test data of glaze surface, as listed in Table 1. Apparently, cracks existed at the interface between the glass phase and rod-like anorthite crystals (Figure 11b and P20 in Table 2) in the body/glaze interlayer (Figure 11a). Stress existed at the interface between anorthite crystals and the glass phase, which was the weak link accompanied by easy generation of cracks under mechanical force [18]. After long-term abrasion by sea wave-borne debris, microcracks further developed and extended to form large cracks, then serving as the invasion channels for foreign pollutants.

Figure 11c displayed serious damages to the glaze layer (No. 3 box in Figure 8a). The EDS data revealed that the filler in the P21 pore (Figure 11d) was iron oxide (P21 in Table 2), suggesting that the glaze had been abraded by sea wave-borne debris before the attachment of coralline algae. The broken pore was full of deposited iron rusts.

Overall, marine plant coralline algae attached to the abraded rough glaze. Three-dimensional interconnected porous channels in coralline algae and microcracks throughout the remains provided the ways for intrusion and sites for deposition. The porous channels of the yellow-white skeleton were filled with iron rusts, sea mud, sea sand and other foreign pollutants, forming the dark composite coagula with cellular structure. 

**Table 2:** EDS analysis results of samples DL-1~3 (wt%).

Figure	Position	Na	Mg	Al	Si	K	Ca	Fe
Figure 2	Rod-like crystals at the breakage position of the glaze (Anorthite, P1)	0.63	0.46	27.64	43.11	1.20	23.03	3.94
	Sill-like crystal clusters composed of needle-like crystals in the naked body (Mullite, P2)	0.44	1.61	32.71	49.98	5.08	1.47	8.72
Figure 4	Residual glaze (P3)		13.54	15.13	51.98	0.49	15.53	3.33
	Deposits in the pores (P4)		8.35	6.45	17.32	0.50	2.49	64.88
	Deposits in the pores (P5)		10.51	7.04	27.65	0.52	1.76	52.52
	Deposits in the pores (P6)		14.46	9.49	36.31	0.71	1.66	37.37
Figure 5	Pollutants deposited on the surface of the inner surface (P7)		11.08	9.65	34.64	0.76	4.68	38.86
Figure 6	Deposits in the pores (P8)		2.00	2.44	89.32			6.23
	Hexagonal crystals (P9)		4.05	26.26	26.16	1.21		42.32
	Short-column crystals (P10)		3.39	23.37	20.43	1.60	0.53	50.68
	Dendritic crystals (P11)		2.42	38.27	1.37			57.93
Figure 10	A-layer coagula (P12)		2.56	6.11	21.92	1.10	5.88	62.44
	B-layer coralline algae skeleton (P13)		12.71		1.61		79.53	6.16
	C-layer coralline algae skeleton (P14)	0.61	8.45		0.50		86.11	4.33
	B-layer filler (P15)		7.61	4.42	24.99	0.64	2.43	59.91
	C-layer filler (P16)		6.45	2.44	23.79	0.67	2.41	64.24
	Filler (P17)		10.23	5.22	31.66	0.93	2.43	49.53
	Coralline algae skeleton + Filler (P18)		8.02	1.49	11.88	0.27	33.15	45.18
Figure 11	Glaze (P19)	0.54	7.09	15.16	51.39	4.56	14.98	6.29
	Crystals in glaze-body interlayer (P20)	2.05	2.23	22.66	50.80	2.46	14.92	4.88
	Foreign pollutants in the pore (P21)	0.88	0.95	2.40	0.22	1.02	94.52	0.88

#### 4.3.3. μ-Raman Analysis Results of the Coagula

**Figure 12:**
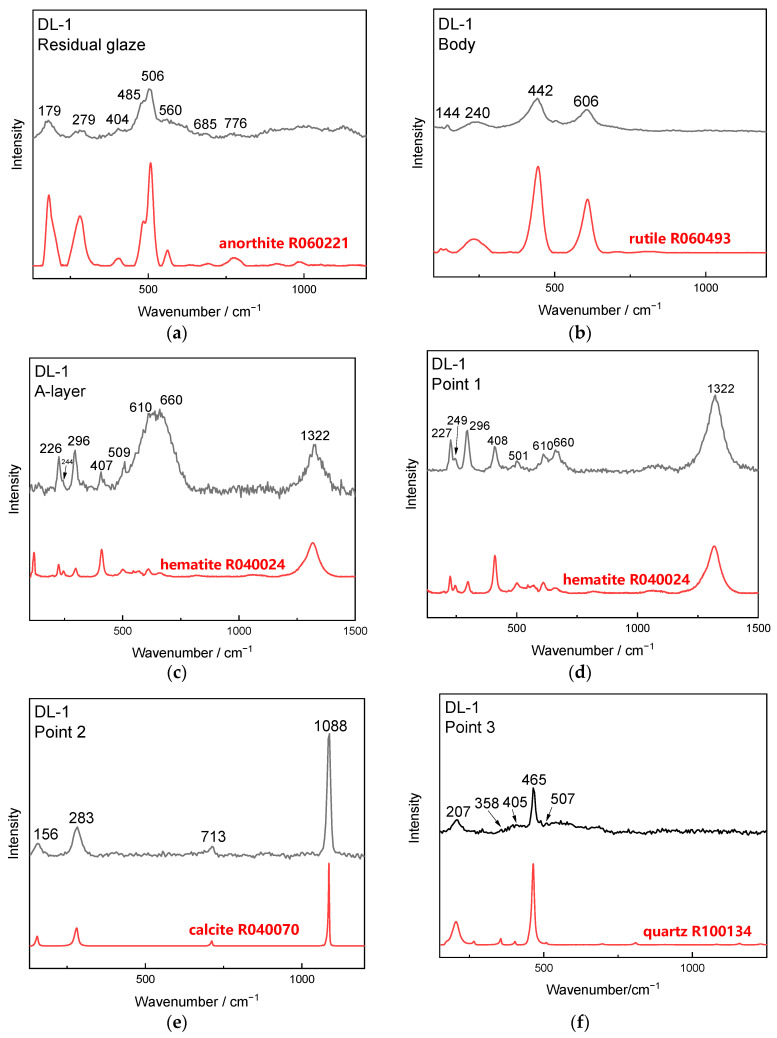

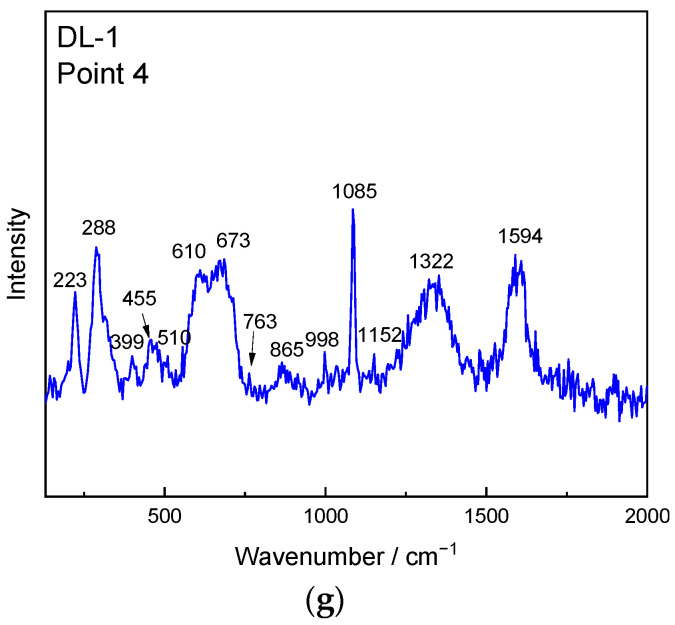
μ-Raman spectra of sample DL-1: (**a**) anorthite in the residual glaze, (**b**) rutile in the body, (**c**) hematite in the A-layer on the cross sections; (**d**–**g**) Point 1–4 on the natural surface of the cellular coagula.

Characteristic positions of sample DL-1 were analyzed using μ-Raman, on the cross-sections of the residual glaze, body and interlayer coagula (A-layer coagula), and on the natural surface of the cellular coagula (Point 1–3), as shown in Figure 12. Several phases, including anorthite (from the residual glaze), rutile (from the body), hematite (from A-layer and Point 1), calcite (from Point 2), and quartz (from Point 3), were identified. Calcite and hematite were located in the yellow-white region and the brick-red region of the cellular coagula surface, respectively. 

The µ-Raman spectra of Point 4 on the surface of the cellular coagula suggested the mixed phases of calcite and hematite (Figure 12e). The cross-sectional diameter of the porous channels in the remains was approximately 10 µm. The diameter of the beam spot in μ-Raman spectra was 25 µm. The test region covered the remains of coralline algae and filler. The EDS micro-area composition test data (P12–P16 in Table 2) demonstrated that the remains of coralline algae were rich in Ca and Mg, and the filler and the interlayer coagula were rich in Fe and Si. Combining the results of μ-Raman and SEM-EDS analysis suggested that the coralline algae remains were mainly composed of calcite, and the filler and the interlayer coagula were composed of hematite and quartz. The iron rusts deposited on the surface pit of the glaze layer below the coralline algae were composed of hematite Fe_2_O_3_ (Figure 11c and P21 in Table 2).

#### 4.3.4. EDXRF and XRD Analysis Results of the Coagula

The EDXRF analysis was also performed on the surface of the cellular coagula. Similar to the EDS results in Table 3, Fe and Si showed the most abundant distribution in the coagula. In combination with the μ-Raman analysis, it was inferred that Si was sourced from gravel sandy sediments on the seabed, with quartz as the main phase, while Fe was sourced from iron rusts. In addition, S, Na, and Cl were also detected in the EDXRF results. To be specific, S was mainly sourced from sulfate in the sea. Under an oxygen-deficient environment, sulfate could be affected by desulfovibrio and transformed into sulfide [12]; Na and Cl, also detected in the coagula, were macro-elements in seawater only next to H and O.

**Figure 13:**
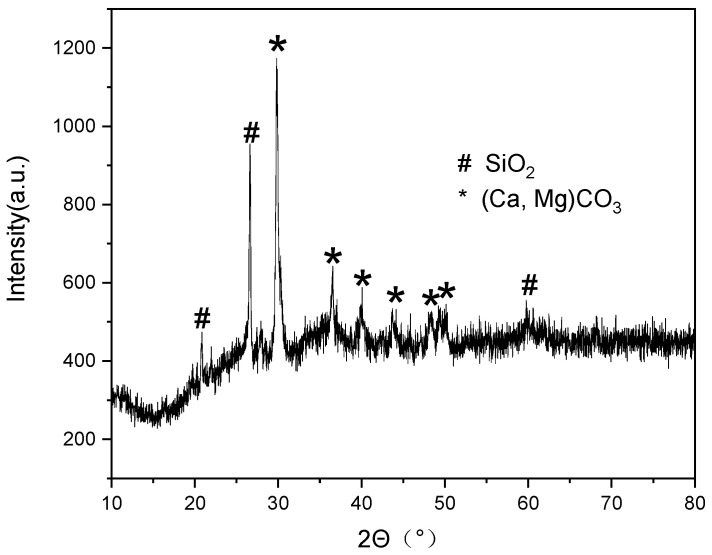
XRD diagram of dark coagula on the outer surface of sample DL-1.

Figure 13 displayed the XRD results on the surface, which showed that the coagula was composed of calcite [(Ca, Mg)CO_3_] and quartz (SiO_2_). This achieved mutual validation with μ-Raman analysis.

### 4.4. Microstructure and Micro-Area Composition of the Altered Glaze

**Figure 14:**
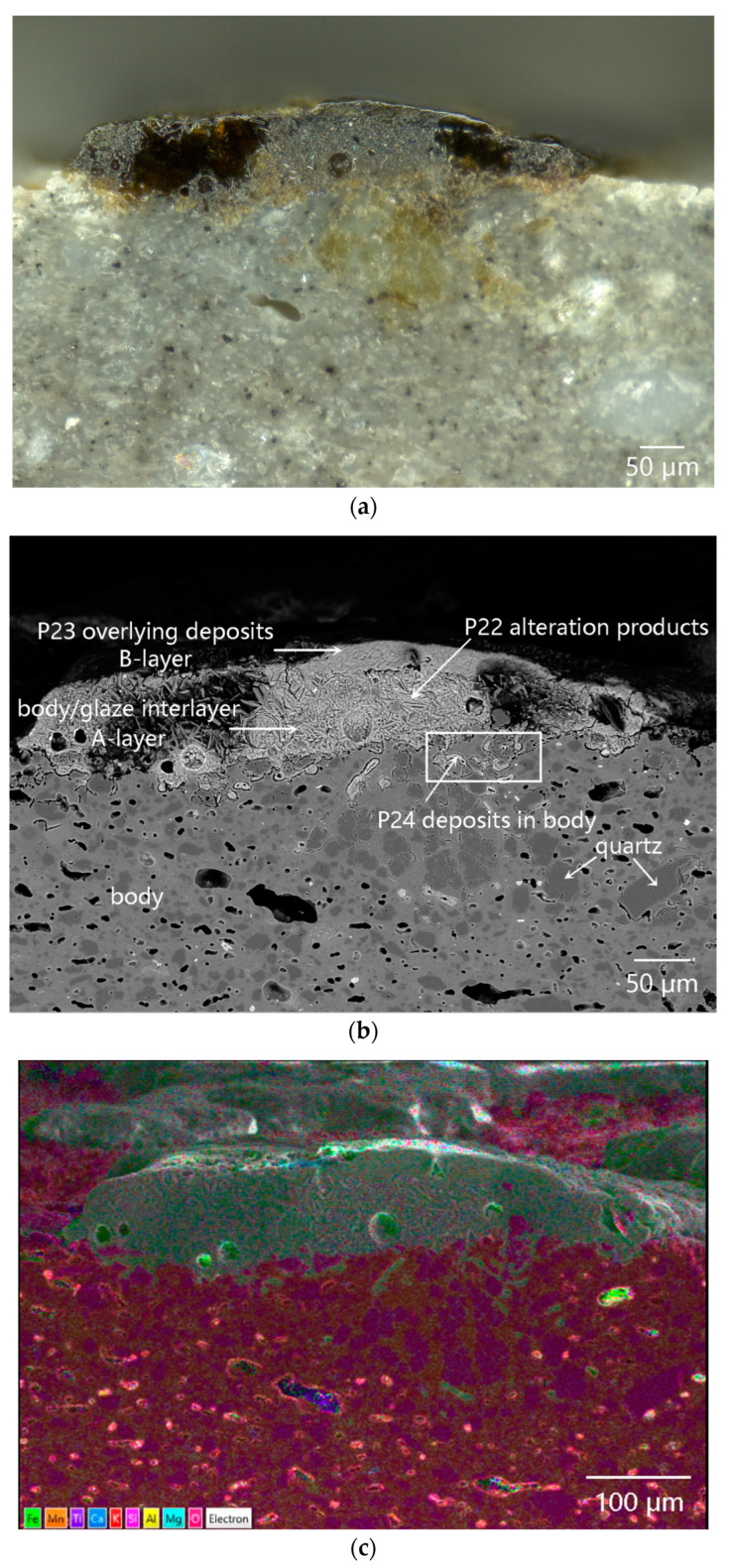
Cross-sectional morphology and element mapping image at the position of residual glaze of sample DL-1: (**a**) OM image, (**b**) SEM image, and (**c**) element mapping images.

**Figure 15:**
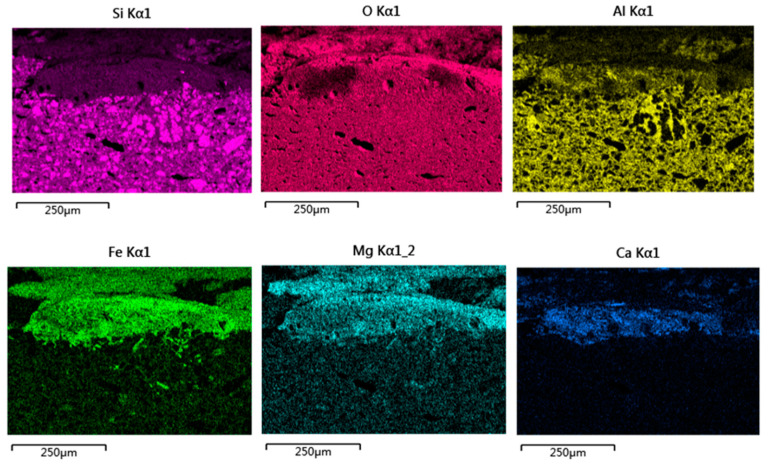
Single-element mapping images of the cross-section at the position of the residual glaze of sample DL-1.

The residual glaze was observed on the outer surface of sample DL-1. The cross-section was then examined using OM and SEM-EDS analysis (Figure 14, Figure 15). As shown in Figure 14a, the residual glaze was rough and uneven. Some dark foreign pollutants below the glaze layer had already entered the body, with a maximum penetration depth of 180 µm. Figure 14b showed that cracks existed at the interface between the residual glaze and the body. Contrary to the residual glaze below the coralline algae (Figure 8b), the glaze-body bonding had already become quite loose, and the glass phase of the glaze showed significant changes. The glaze at the surrounding position had already been completely peeled off with the exposed body.

Cross-sectional morphology and element mapping images showed that the body/glaze interlayer, including a lot of anorthite crystals (A-layer in Figure 14b), with a thickness of approximately 80–90 µm, could be clearly observed. The glass phase around the crystals is rich in Fe, Si and O, and a small amount of Mg and Al (A-layer in Figure 14b, P22 in Table 4), which is quite different from the chemical composition of the glass phase originally wrapping the anorthite crystals. The B-layer above the interlayer (Figure 14b), with a thickness of approximately 25–30 µm, was similar to that filled among crystals in chemical composition (P23 in Table 4). However, the B-layer contained more Mg and less Al. The box in Figure 14b also showed that the cracks in the body were filled with substances with low contrast. The EDS data (P24 in Table 4) indicated the similarity with P22 (in Table 4) in chemical composition and a higher content of Fe.

Referring to the position of anorthite crystals, it was concluded that the original glass phase in the glaze underwent changes under a specific marine environment and was replaced by newly-generated alteration products. Meanwhile, anorthite crystals were reserved. The high content of Fe in the alternation product (up to 47 wt%) proved that foreign iron rusts participated in the alteration reaction. The EDS data indicated that the overlying deposits included alteration products and more Mg deposited from seawater. The seawater carried alteration products and foreign pollutants, which were then transported to the interface between the body and the interlayer via the damaged interlayer (the box in Figure 14b) and finally deposited in the cracks of the body.

## 5. Discussion

According to previous studies, the glazes of the ceramic wares stacked in a shipwreck cabin may undergo chemical alterations such as ion exchange and hydrolysis in the marine environment [17,18]. Most of them looked bright as new after being salvaged out of water, while a small proportion of them became rough and matt. However, the situation with the sauce-glazed ware DL is quite different. Because the original stacking of the ceramic wares in the cabin was destroyed by external forces, DL was carried by ocean currents to the seabed. The abrasion from sea wave-borne debris may have been the primary trigger for DL’s degradation. Different positions on the surface of the ware showed mechanical damage to varying degrees. Some partly-damaged glaze could still be found below the coralline algae remains attached to the outer surface, suggesting that coralline algae were attached to the surface before the complete spalling of the glaze layer on the outer surface.

Crust coralline algae attached to the surface of DL could be classified as a type of coralline algae in Corallinales, Rhodophyta, most of which were attached to the hard matrix. Before the attachment of coralline algae, the surface of DL had already undergone the first two steps of marine biological fouling. First, proteins and polysaccharides sourced from the excrement of living creatures or the decomposition products from dead creatures were enriched on the surface of DL, accompanied by the formation of the conditioning film. Next, creatures such as bacteria and single-cell microalgae were attached, which secreted and produced extracellular polymeric substances, forming the microbial biofilm [22]. Based on this, coralline algae spores were captured by the biological film on the surface of DL [23], and grew constantly with the biological film as the nutritional source. The hard calcareous skeletons formed by the bio-mineralization process could resist the direct impact of mechanical action on the glaze layer, preventing further damage. The anorthite/glass phase interface and the body/interlayer interface under stress concentration were subjected to external impact, resulting in the initiation and development of microcracks. Under external mechanical action, the microcracks induced by phase transformation developed around artificially-added quartz sands and grew to large cracks. These large cracks were not only interconnected, but also connected with the pores in the body [4].

After the degradation of the cytoplasm of coralline algae, the open-type cellular skeleton remains with a pore size of less than 10 µm were formed. Seawater carried the inorganic pollutants, such as sea sands and iron rusts, and invaded the coralline algae remains via hollow porous channels in the skeleton, further entering into the glaze and the body via the cracks. Finally, complex coagula with the unique lamellar structure were formed on the surface of DL.

The surface without the attachment of marine organisms was seriously damaged, and the glaze on the outer surface almost completely peeled off. A large number of open pores, crevasses, and cracks were produced on both the inner and outer surfaces, which then became the preferential habitats for fouling organisms in the sea. These also provided intrusion channels and deposition sites for some foreign pollutants, including fine sea sands and iron corrosion products, further contributing to the degradation.

The electrochemical reaction of iron corrosion in the sea was as follows.

The reaction at the cathode was expressed as:

(1) $$\frac{1}{2}O_{2}+{\;H}_{2}O+2e^{-}\;\to \;2{\mathrm{OH}}^{-}$$

The reaction at the anode was expressed as:

(2) $$\mathrm{Fe}\;\to {\;\mathrm{Fe}}^{2+}+2e^{-}$$

As the corrosion continued, the following secondary reaction happened [24]:

(3) $${\mathrm{Fe}}^{2+}+2{\mathrm{OH}}^{-}\to \mathrm{Fe}{\left(\mathrm{OH}\right)}_{2}$$

(4) $$4\mathrm{Fe}{\left(\mathrm{OH}\right)}_{2}+O_{2}+2H_{2}O\to 4\mathrm{Fe}{\left(\mathrm{OH}\right)}_{3}$$

(5) $$4\mathrm{Fe}{\left(\mathrm{OH}\right)}_{2}+O_{2}\to 4\gamma -\mathrm{FeOOH}+2H_{2}O$$

(6) $$2\mathrm{Fe}{\left(\mathrm{OH}\right)}_{3}\to {\mathrm{Fe}}_{2}O_{3}+3H_{2}O$$

A large number of Cl^-^ ions in seawater could have reacted with Fe^2+^ to produce soluble FeCl_2_ and diffused toward the surrounding environment. Therefore, despite the detection of the existence of hematite on the surface of DL, a wide variety of iron corrosion products existed in the sea, mainly including Fe(OH)_3_ [in Equation (4)] andγ-FeOOH [in Equation (5)]. Complex iron corrosion products were deposited and penetrated into the glaze layer. Part of these products was redissolved and released in the form of soluble salts, such as Fe^2+^ and Fe^3+^, and reacted with the glass phase in the glaze. Anorthite crystals in the altered glaze layer remained in situ, while the glass phase was replaced by the alteration products. Meanwhile, the sauce-glazed ware was in a weakly alkaline (pH = 8.16 in Ref. [25]) marine environment, and the bio-mineralization of the attached coralline algae led to an increase in the pH value of the microenvironment [26], both promoting the hydrolysis of the Si-O-Si networks in the glaze. The hydrolysis products reacted with the iron corrosion products to form a new phase, iron silicate [27,28].

As iron silicate was constantly produced, Si in the surrounding environment of sample DL was never saturated and the glass phase was constantly hydrolyzed, finally leading to the alteration and disappearance of the whole glass phase. On account of hydrolysis and de-alkalization, Ca in the original glass phase was consumed, and Mg^2+^ was deposited in the alteration layer in the form of Mg(OH)_2_ [29] [in Equation (7)]:

(7) $${\mathrm{Mg}}^{2+}+2{\mathrm{OH}}^{-}\to \mathrm{Mg}{\left(\mathrm{OH}\right)}_{2}↓$$

**Figure 16:**
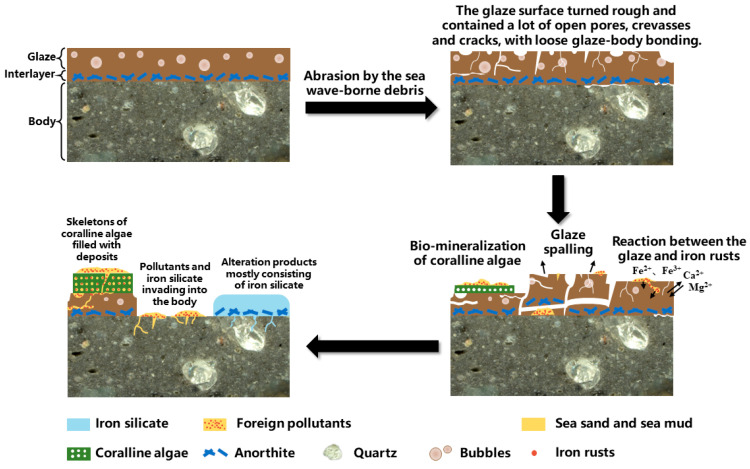
Illustration of the degradation process of the sauce-glazed ware DL.

Based on the above discussion, the degradation process of DL was plotted, as shown in Figure 16. Firstly, pores and cracks were formed on the glaze surface after abrasion by the sea wave-borne debris, leading to the poor preservation state of the ware. Coralline algae were tightly attached to the rough glaze surface. The calcareous skeleton with micron-sized pore channels was filled with foreign pollutants such as iron rusts, sea sands, and sea mud, preventing further damage to the residual glaze. The spalling of the glaze resulted in the exposure of body, and foreign pollutants deposited on the body surface. Meanwhile, iron rusts also reacted with the glaze and caused the chemical alteration. Anorthite crystals in the body/glaze interlayer did not participate in the reaction but remained at the original position. The alteration products, mostly consisting of iron silicate, gradually replaced the original glass phase and invaded into the body via pores and cracks.

## 6. Conclusions

In this study, the degradation mechanism of the sauce-glazed ware DL in the marine environment was investigated by analyzing its complex degradation morphologies. The final preservation state of the ware was the result of the combined action of mechanical damage, marine bio-fouling, and chemical alteration. First, the ware underwent mechanical force induced by the motion of waves, which significantly reduced its corrosion resistance. Abrasion by the sea wave-borne debris was regarded as the primary factor leading to the poor preservation state of the ware, which also served as the precondition for the subsequent attachment of marine organisms and the deposition of inorganic pollutants. Coralline algae were tightly attached to the rough glaze surface. The calcareous skeleton with micron-sized pore channels provided sites for the deposition of foreign pollutants such as iron rusts, sea sands, and sea mud. The calcareous skeleton also contributed to resisting the impacts of wave motions and slowed down the degradation process. Meanwhile, inorganic pollutants represented by iron rusts were also involved in the degradation. The pollutants deposited on surface pits and cracks resulted in stress to the glaze and the glaze/body interface, causing further cracking and even spalling of the glaze. Iron rusts also reacted with the glaze glass phase and caused the chemical alteration. The alteration products were mostly iron silicate. Anorthite crystals in the body/glaze interlayer did not participate in the reaction but remained at the original position. The alteration products gradually replaced the original glass phase and invaded into the body via pores and cracks, thereby accelerating the degradation process. 

## Figures and Tables

**Table 1:** EDXRF analysis results of the major and minor elements of the body and glaze (wt%).

DL-1	Mg	Al	Si	K	Ca	Ti	Fe	Mn	P	S
Body	0.26	17.47	64.59	6.52	0.59	1.68	8.85	0.04		
Glaze (polluted and altered)	7.07	3.73	15.65	0.40	27.23	0.12	38.44	6.75	0.29	0.32

**Table 3:** EDXRF analysis results of the dark coagula on the outer surface of sample DL-1.

	Na	Mg	Al	Si	S	Cl	K	Ca	Mn	Fe
Dark coagula	1.03	5.09	7.96	21.46	1.78	2.19	1.49	2.76	3.13	52.14

**Table 4:** EDS analysis results of the alteration products and deposits (wt%).

Figure	Position	Mg	Al	Si	K	Ca	Fe
Figure 14	Alteration products in the interlayer (P22)	8.98	8.15	31.05	0.55	4.11	47.16
	Overlying deposits (B-layer, P23)	11.29	4.58	32.00		1.70	50.42
	Deposits in the body (P24)	8.25	7.29	29.79	0.77	1.16	52.72

## Data Availability

Data sharing not applicable.
